# Bacterial Communities of House Flies from Dairy Farms Highlight Their Role as Reservoirs, Disseminators, and Sentinels of Microbial Threats to Human and Animal Health

**DOI:** 10.3390/insects15090730

**Published:** 2024-09-22

**Authors:** Saraswoti Neupane, Yoonseong Park, D. Wes Watson, Rebecca T. Trout Fryxell, Edwin R. Burgess, Dana Nayduch

**Affiliations:** 1 Department of Entomology, Kansas State University, Manhattan, KS 66506, USA; ypark@ksu.edu; 2 Department of Entomology and Plant Pathology, North Carolina State University, Raleigh, NC 27695, USA; wwatson@ncsu.edu; 3 Department of Entomology and Plant Pathology, University of Tennessee, Knoxville, TN 37996, USA; rfryxell@utk.edu; 4 Department of Entomology and Nematology, University of Florida, Gainesville, FL 32611, USA; edwinburgess@ufl.edu; 5 Arthropod-Borne Animal Diseases Research Unit, Center for Grain and Animal Health Research, USDA-ARS, Manhattan, KS 66502, USA

**Keywords:** house fly, bacterial community, diversity, bacterial pathogens, dairy cattle farm, cattle manure, rumen-associated bacteria

## Abstract

**Simple Summary:**

Adult house flies found in livestock operations pose significant challenges not only as nuisance pests but also as vectors of a wide range of microbial pathogens affecting both humans and animals. This study investigated bacterial community composition and diversity in female house flies and cattle manure collected from dairy cattle farms across three US states. The findings revealed that flies carried bacterial taxa acquired from their environment, capturing the entire bacterial microbiome from manure along with other taxa likely acquired from the animals. Taxa of potential pathogens were highly abundant in house flies, suggesting that flies pose a biosecurity risk in spreading microbes with human and animal importance both within dairy operations and potentially off-site.

**Abstract:**

Adult house flies (*Musca domestica* L.) inhabiting dairy farms not only are nuisance pests but also harbor and disseminate bacteria. We examined the bacterial community composition, diversity, environmental sources, and prevalence in individual adult female house flies and cattle manure samples collected monthly from Florida, North Carolina, and Tennessee dairy farms between May and August 2021. Individual house flies carried diverse bacterial communities, encompassing all bacterial taxa (100%) identified across manure samples, and additional species likely acquired from the animals. Bacterial community assemblage in house flies and manure samples within farms varied by month. Some taxa were differentially associated with either house flies (*Corynebacterium*, *Acinetobacter*, and *Staphylococcus*) or manure samples (*Treponema*, *Succinivibrio*, and Clostridia). House fly bacterial communities mostly contained specialist species originating from manure, with several taxa (*Escherichia, Corynebacterium*, *Turicibacter*) being potential pathogens of livestock and humans. These findings further support the role of house flies as carriers of cattle-associated bacteria, including pathogens, and their potential for disseminating these microbes among cattle and to neighboring environments. Since their bacterial communities provide a snapshot of their surrounding environment, house flies also serve as effective sentinels in xenosurveillance strategies.

## 1. Introduction

Trophic behaviors of adult house flies (*Musca domestica*, L.) drive their frequent contact with microbe-rich substrates including human and animal waste, wounds, discharge, and decaying organic matter. As a result, flies acquire, carry, and disseminate diverse microbial communities, including hundreds of microbial taxa (e.g., bacteria, viruses, fungi, protists, and nematodes) of veterinary and medical importance (reviewed in [1,2,3,4]). Numerous studies have shown adult house flies not only carry but also can transmit bacterial pathogens that cause diseases in domestic animals and/or humans [5,6,7,8,9,10]. In terms of cattle, adult house flies captured in close proximity to cows with clinical symptoms of mastitis carried the bacterial pathogen *Corynebacterium pseudotuberculosis* [6]. Similarly, flies collected from outside “sick pens” containing beef calves with visible bovine respiratory disease (BRD) symptoms harbored bacterial pathogens of BRD, *Mannheimia haemolytica*, *Pasteurella multocida*, and *Histophilus somni* [10]. Other studies have provided direct evidence of bacterial pathogen transmission by house flies to domestic animals. For example, house flies contaminated with *C. pseudotuberculosis* Biovar *equi* released near healthy confined horses caused the development of clinical symptoms of abscesses [9]. Therefore, linking microbial communities in house flies to livestock operations and understanding factors affecting microbial communities is a logical next step toward improving disease surveillance and mitigating the transmission risk of potential fly-borne diseases of livestock.

Adult house flies in confined dairy cattle farms are a biosecurity risk due to their unrestricted access to farm waste such as cattle manure as well as animal excretions and wounds, which are ample sources of microbes. Microbial communities of cattle manure are composed of both prokaryotic (archaea, bacteria) and eukaryotic (fungi, protists) microorganisms [11,12]. Cattle manure contains diverse fecal bacterial communities, including pathogens such as *Escherichia coli* [13], *Campylobacter jejuni* [14], and *Salmonella* spp. [13]. Cattle fecal bacterial communities are influenced by both biotic (cattle physiology, health status, age) and abiotic (feed, farm management) factors [12,15,16,17]. The community composition of cattle manure varies between farms [18] and individual animals [19]. Interestingly, bacterial communities in adult house flies collected from livestock and other animal farms have commonalities with respective communities of co-located animal dung, although this knowledge is limited [20,21].

Individual house flies can carry thousands of microbial taxa within their bodies [20,21,22,23,24]. At cattle farms in particular, individual house flies have been shown to harbor highly diverse but uniquely composed bacterial communities. However, a number of bacterial taxa, including potential pathogens like *Corynebacterium*, *Clostridium,* and *Escherichia*–*Shigella,* were highly prevalent and part of the core bacterial communities of house flies [20,21,22,24]. A high prevalence of potential pathogens suggests an increased risk of flies spreading those bacteria among animals or even off-site to human habitats or other farms. Unraveling bacterial community composition and ecology in house flies and determining suspected microbial sources from their habitats are essential steps towards improving our understanding of the house flies’ role in spreading bacteria that pose threats to animal health and food safety.

Our previous studies revealed that the composition and diversity of bacterial communities in individual adult female house flies from beef and dairy cattle farms are associated with the farm environment in which they reside and vary both temporally and spatially [21]. Therefore, in this study, we expanded our aims to better understand the roles that house flies play in carrying and disseminating bacterial communities in confined dairy farms. We characterized bacterial communities from individual adult female house flies and cattle manure samples concomitantly by sampling six dairy cattle farms situated in three states (two farms per state) in the eastern USA once per month for four months and evaluated whether female house flies acquire their bacterial communities from co-located manure samples. We also assessed the influence of sampling month and farm on the abundance of bacterial pathogens, bacterial community composition, and diversity.

## 2. Materials and Methods

### 2.1. Sample Collection Sites

This study was conducted in six dairy farms located in three U.S. states. A pair of dairy farms in each state, with the following cities, served as collection sites: Florida, Hawthorne (FLH) and Gainesville (FLU); Tennessee, London (TNL) and Philadelphia (TNP); North Carolina, Chapel Hill (NCC) and Raleigh (NCR) (Table S1). The distance between pairs of farms in each state was 10 to 46 km, whereas the distance between farms across states ranged from 467 to 1155 km. Metadata from sample collection sites, including cattle breed, age, cattle diet, presence/absence of nearby farms, manure management, and antibiotic and pesticide use, are summarized in Table S1.

### 2.2. Sample Collection and Processing

Samples of adult female house flies (n = 10 per farm/date) and manure (n = 3 per farm/date) were collected concomitantly from each farm once a month (four times) between May and August 2021 (Table S1) and processed as described previously by Neupane et al. [21]. It is important to note that our decision to sample only adult female flies was based on our previous study, which demonstrated that individual adult female house flies harbor a greater abundance of culturable bacteria compared to male flies [25]. All samples were shipped to the laboratory at the United States Department of Agriculture, Agricultural Research Service (USDA-ARS), in Manhattan, Kansas, and subsequently stored at +4 °C (for <1 month storage) and/or −20 °C (for >1 month storage) until DNA extraction, which occurred after all samples were collected.

### 2.3. DNA Extraction, Library Preparation, Sequencing, and Analysis

Genomic DNA was extracted from individual house flies, 0.25 g of manure sample, positive control sample (laboratory culture of *Staphylococcus aureus*), and negative control sample (DNA/RNA shield) using ZymoBIOMICS DNA Kits (Zymo Research, Irvin, CA, USA) as described in the manufacturer’s protocol. DNA quality and quantity were determined as described previously [21] and DNA samples were submitted to the Genome Sequencing Core, University of Kansas, for amplicon sequencing of the bacterial rRNA gene (V3–V4 region) on the Illumina MiSeq.

Demultiplexed raw sequence data were analyzed in Mothur (version 1.48.0, [26]) as described previously [27]. Briefly, high-quality sequences with 97% sequence similarity were assembled into an operational taxonomic unit (OTU). Representative sequences of OTUs were aligned and a phylogenetic tree was constructed. Taxonomy for representative sequences of each OTU was assigned as described previously [27]. OTUs assigned to the same phylum and/or genus were grouped to obtain abundances of bacterial communities at the phylum and/or genus level, respectively. Potential bovine and human pathogenic taxa (genera) were selected based on previously known pathogenic genera. Bacterial OTUs with ≥0.1% relative abundance and present in ≥80% of fly or manure samples were considered core bacterial communities of fly or manure samples. Due to a low number of sequences remaining after quality filtering (<2100 sequence reads), four samples (two flies from each farm in FLU and TNL) were removed from the dataset. The final data and phylogenetic tree were used for further statistical analysis.

### 2.4. Statistical Analysis

Statistical analyses were performed in the R statistical program (version 4.3.1, [28]) with vegan (version 2.6-4, [29]), emmeans (version 1.8.7, [30]), ape [31], microeco [32], EcolUtils (version 0.1, [33]) and ggplot2 (version 3.3.3, [34]) packages. Linear discriminant analysis standard effect size (LEfSe) was used to determine if bacterial OTUs were differentially abundant in sample types and/or in farms within sample type. OTUs with LDA scores >2 and *p* < 0.05 were considered differentially abundant in an environment. Fifty of these differentially abundant taxa were plotted in bar graphs. Further, differential features in the taxonomic tree were determined for the 200 most abundant OTUs for sample type and/or farms within sample type, and 50 taxa with the most differential features in the taxonomic tree were plotted in cladograms. Bacterial alpha diversity (Shannon diversity index) was calculated for each sample. A general linear model was applied to determine the effects of sample type and farm, or farm and sampling month, on the Shannon diversity index of all samples (both fly and manure), or house flies only and manure samples only. In the model, the Shannon diversity index was the response variable, and sample type, farm, and sample type-by-farm interaction, or farm, sampling month, and farm-by-sampling month interaction were predictor variables. Subsequently, estimated marginal means (EMMs) were calculated and pairwise comparisons of EMMs were made to determine the significant differences between sample types, among farms, or sampling months. The *p*-values were Tukey-adjusted and *p* < 0.05 was considered significant. Using community abundance data (either fly or manure), OTUs were further classified into generalists, specialists, or neutral groups. The classification was based on deviation from niche width index from null values determined by permutation algorithms for community matrices. Levin’s niche width index for each OTU was calculated for 1000 null matrices and compared to the deviation from mean index value and confidence intervals to determine generalists, specialists, and neutral groups. Further, the effects of farm and sampling month on the relative abundance of each group were analyzed using linear models as described above (see Shannon diversity index). The effects of farm, sampling month, and farm-by-sampling month interactions on relative abundances of major phyla and potential pathogenic genera in each sample type were investigated using a general linear model as described above. The differences in abundances across sampling months or farms were also determined as described above. Principal coordinate analysis (PCoA) was used to evaluate the bacterial assemblage in individual house flies and manure samples within each farm. For this, the OTU abundance dataset was Hellinger-transformed and then the Bray–Curtis dissimilarity matrix was calculated, after which PCoA was performed. The bacterial community composition was visualized by plotting the first two PCoA axes. To further evaluate the differences in community assemblage (community composition) among sample types and/or sampling months, permutation-based multivariate analysis (adonis) was performed with dissimilarity matrices for each farm.

## 3. Results

### 3.1. Bacterial Communities in House Fly and Manure Samples

**Figure 1:**
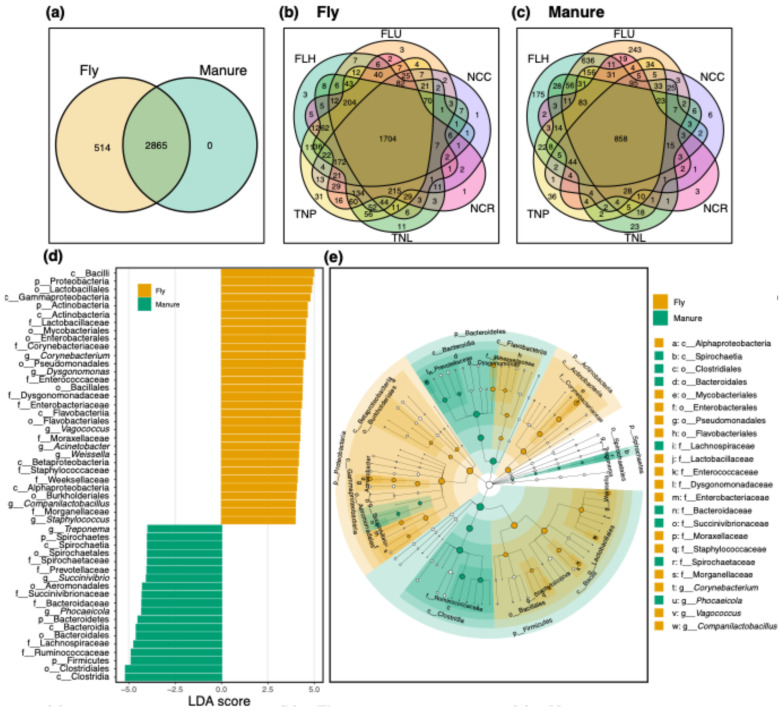
Bacterial communities in house fly and manure samples associated with dairy cattle farms from three states (Florida: FLH and FLU; Tennessee: TNL and TNP; North Carolina: NCC and NCR). Shared and unique bacterial OTUs (operational taxonomic units) observed in (**a**) overall, between house flies and manure irrespective of location and sampling time; (**b**) flies, among locations irrespective of sampling time; and (**c**) manure, among locations irrespective of sampling time. Overlapping sections represent shared bacterial diversity (i.e., observed number of OTUs) between indicated samples, while non-overlapping sections show OTUs unique to the sample type (**a**) or location (**b**,**c**). (**d**) Linear discriminant analysis (LDA) effect size (LEfSe) scores indicating differential associations of bacterial taxa in house fly and manure samples, irrespective of location. (**e**) Cladogram depicting differential features in the taxonomic tree of bacterial taxa between house fly and manure samples from dairy farms. Size of the node circles represents the relative abundance of the bacterial taxon. p = phylum; c = class; o = order; f = family; g = genus. Additional descriptions of farms can be found in the main text.

**Figure 2:**
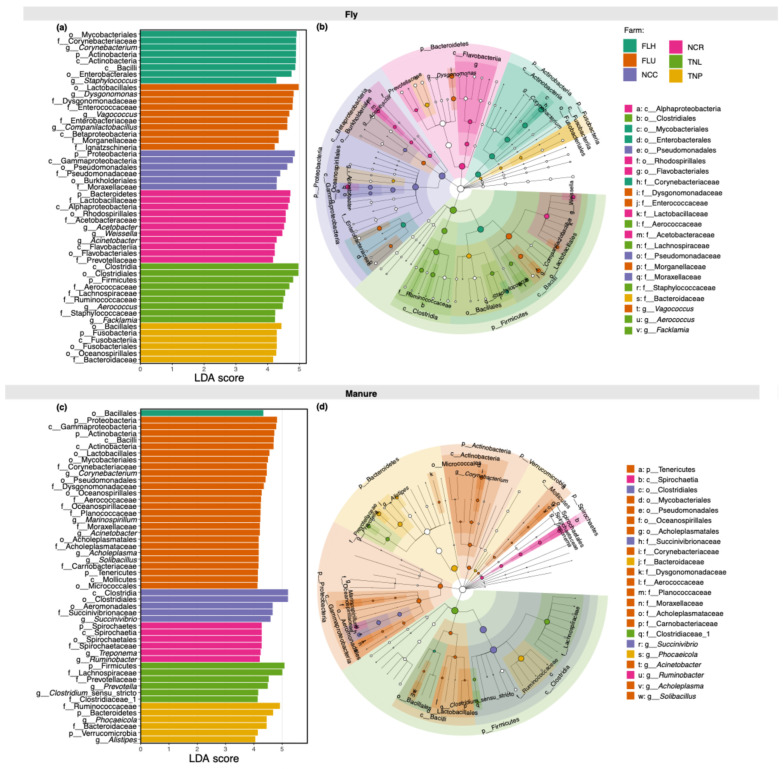
Differentially abundant bacterial taxa in house fly or manure samples from dairy cattle farms from three states (Florida: FLH and FLU; Tennessee: TNL and TNP; North Carolina: NCC and NCR). Linear discriminant analysis (LDA) effect size (LEfSe) scores show bacterial taxa differentially associated with (**a**) house fly and (**c**) manure samples in each farm. Cladograms for (**b**) house fly and (**d**) manure showing the differential features in the taxonomic tree of bacterial taxa among farms. Size of the node circles represents the relative abundance of the bacterial taxon. p = phylum; c = class; o = order; f = family; g = genus. Additional descriptions of farms can be found in the main text.

Overall, bacterial communities in both house flies and manure samples from dairy cattle farms comprised 3379 OTUs, and all bacterial OTUs associated with manure samples were found in house fly samples (Figure 1a). Approximately 15% of OTUs (5.85% of total sequence reads) were unique to house fly samples. Although house flies harbored all OTUs associated with manure samples, several OTUs were differentially associated with fly and/or manure samples (Figure 1d,e). For example, OTUs classified as Bacilli, *Corynebacterium*, Enterobacteriaceae, *Dysgonomonas*, Moraxellaceae, *Acinetobacter*, *Weissella,* and *Staphylococcus* (LDA scores: 4.96, 4.49, 4.32, 4.36, 4.22, 4.17, 4.14, and 3.95, respectively; all *p* < 0.0001) were more closely associated with fly samples, while Clostridia, *Treponema*, *Phocaeicola*, *Succinivibrio*, Prevotellaceae, and Bacteroidaceae (LDA scores: 5.18, 3.96, 4.30, 4.02, 4.01, and 4.29, respectively; all *p* < 0.0001) were more closely associated with manure samples (Figure 1d,e). However, in individual farms, house flies shared only 40.8–71.7% of OTUs, whereas 16.3–57.1% were unique to flies and 0.5–14.0% were unique to manure samples (Figure S1a–f). Additionally, 50.43% of bacterial OTUs in house fly samples and 29.95% of bacterial OTUs in manure samples were shared across farms (Figure 1b,c). Various levels of differential associations between bacterial taxa within each farm and within a sample type (fly or manure) were observed (Figure 2a–d). For example, at the FLH farm, fly samples were more likely to have *Corynebacterium*, *Staphylococcus,* and Mycobacteriales (LDA scores: 4.87, 4.29, and 4.89, respectively; all *p* < 0.0001), while manure samples were more likely to have Bacillales (LDA score: 4.32; *p* < 0.0001). Similarly, at the FLU farm, fly samples were more likely to have Enterobacteriaceae, *Ignatzschineria*, *Vagococcus,* and Enterococcaceae (LDA scores: 4.76, 4.18, 4.65, and 4.78, respectively; all *p* < 0.0001), while Proteobacteria, *Corynebacterium*, Pseudomonadales, and Moraxellaceae (LDA scores: 4.81, 4.45, 4.39, and 4.17, respectively; all *p* < 0.0001) were more likely associated with manure samples. At the NCC farm, Pseudomonadaceae, Moraxellaceae, and Proteobacteria (LDA scores: 4.37, 4.29, and 4.85, respectively; all *p* < 0.0001) were more likely associated with fly samples, while Clostridia and Aeromonadales (LDA scores: 5.20 and 4.67, respectively; all *p* < 0.0001) were more likely associated with manure samples. At the NCR farm, fly samples were more likely to contain Bacteroidetes, *Acinetobacter,* and *Weissella* (LDA scores: 4.74, 4.31, and 4.46, respectively; all *p* < 0.0001), while manure samples were more likely to have Spirochaetes and *Treponema* (LDA scores: 4.26 and 4.22; *p* = 0.002 and 0.003, respectively). At the TNL farm, fly samples were more likely to have Clostridia, *Aerococcus,* and Staphylococcaceae (LDA scores: 4.97, 4.45, and 4.26, respectively; all *p* < 0.0001), while manure samples were more likely to have Firmicutes, *Prevotella,* and *Clostridium* sensu stricto (LDA scores: 5.08, 4.50, and 4.14, respectively; all *p* < 0.0001). At the TNP farm, fly samples were more likely to have Bacillales, Bacteroidaceae, and Fusobacteria (LDA scores: 4.49, 4.20, and 4.24, respectively; all *p* < 0.0001), while manure samples were more likely to have Bacteroidetes, *Phocaeicola,* and *Alistipes* (LDA scores: 4.62, 4.42, and 4.03, respectively; all *p* < 0.0001).

Bacterial OTUs with ≥0.1% of total frequency and observed in ≥80% of either fly or manure samples were considered core communities of fly or manure samples. In fly samples, there were 55 such OTUs and some of them were classified as *Corynebacterium*, *Acinetobacter*, *Staphylococcus*, *Escherichia*–*Shigella*, *Clostridium* sensu stricto, *Enterococcus*, *Providencia*, *Phocaeicola*, *Succinivibrio*, and *Turicibacter,* while in manure samples there were 106 OTUs, including OTUs classified as *Phocaeicola*, *Succinivibrio, Turicibacter, Clostridium* sensu stricto, *Romboutsia*, and Ruminococcaceae.

### 3.2. Abundance of Potential Pathogens in House Fly and Manure Samples

**Figure 3:**
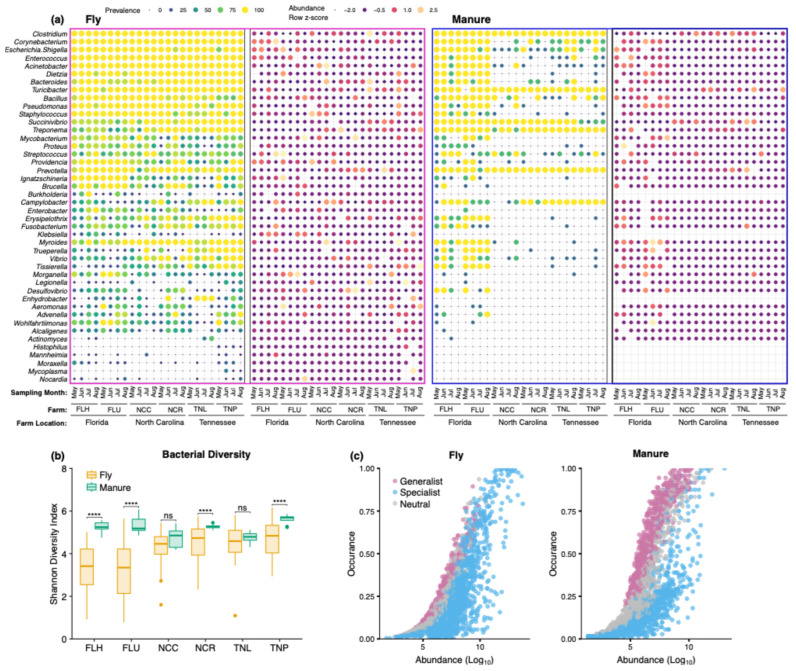
Communities and diversity of bacteria in house fly and manure samples associated with dairy cattle farms from three states (Florida: FLH and FLU; Tennessee: TNL and TNP; North Carolina: NCC and NCR). (**a**) Prevalence (%) and abundance (row z-score) of potential pathogenic taxa identified in flies (n = 10, except FLU and TNL, n = 8) and manure (n = 3) samples per sampling time (May–Aug) and farm. (**b**) Overall bacterial Shannon diversity index in house fly (n = 40, except FLU and TNL, n = 38) and manure (n = 12) samples associated with each farm, irrespective of sampling time. Plot showing the median and interquartile ranges (25th–75th percentile; boxes) with whiskers representing upper and lower values, respectively, and outlier points. Asterisks indicate significant differences between sample types (****, *p* < 0.000; ns, non-significant). (**c**) Occurrence and abundance of bacterial species that are generalists, specialists, or neutral in house fly and manure samples across all sites and collection times. Additional descriptions of farms can be found in the main text.

Several bacterial taxa with the potential to cause diseases in livestock and/or humans were detected in both fly and manure samples. Taxa such as *Clostridium*, *Turicibacter, Prevotella*, *Succinivibrio,* and *Treponema* were highly prevalent (>80% of samples) and were detected in both fly and manure samples associated with dairy farms (Figure 3a and Figure S2). *Clostridium*, *Corynebacterium*, *Acinetobacter*, *Enterococcus,* and *Turicibacter* were both prevalent and abundant in flies (prevalence: all 100%; mean relative abundance: 0.85, 8.06, 3.82, 1.21, and 0.62%, respectively; Figure 3a and Figure S2). Some of these taxa were also members of core bacterial communities of house fly and manure samples (described above).

The sample type-by-farm interaction significantly affected the relative abundances of some of these potential pathogenic taxa such as *Clostridium* (F_(5, 296)_ = 7.88, *p* < 0.0001), *Corynebacterium* (F_(5, 296)_ = 3.02, *p* = 0.01), *Turicibacter* (F_(5, 296)_ = 2.73, *p* = 0.02), *Pseudomonas* (F_(5, 296)_ = 2.97, *p* = 0.01), and *Prevotella* (F_(5, 296)_ = 10.63, *p* < 0.0001), while several taxa were significantly influenced by sample type, including *Escherichia*–*Shigella* (F_(1, 296)_ = 14.81, *p* = 0.0001), *Enterococcus* (F_(1, 296)_ = 25.71, *p* < 0.0001), *Acinetobacter* (F_(1, 296)_ = 15.08, *p* = 0.0001), *Bacteroides* (F_(1, 296)_ = 8.46, *p* = 0.004), and *Providencia* (F_(1, 296)_ = 4.52, *p* = 0.03) (Table S2). Significantly lower relative abundances of *Clostridium* (t = −9.31, *p* < 0.0001), *Turicibacter* (t = −4.93, *p* < 0.0001), and *Prevotella* (t = −4.79, *p* < 0.0001) were detected in fly samples compared to manure samples, whereas the relative abundances of *Corynebacterium* (t = 6.28, *p* < 0.0001), *Escherichia*–*Shigella* (t = 3.85, *p* = 0.0001), *Enterococcus* (t = 5.08, *p* < 0.0001), *Pseudomonas* (t = 3.19, *p* = 0.002), *Bacteroides* (t = 2.90, *p* = 0.004), *Acinetobacter* (t = 3.88, *p* = 0.0001), and *Providencia* (t = 2.13, *p* = 0.03) were significantly higher in fly samples than in manure samples. 

Within fly samples, the farm-by-sampling month interaction significantly influenced the relative abundances of several potential pathogens such as *Acinetobacter* (F_(15, 212)_ = 2.15, *p* = 0.009), *Dietzia* (F_(15, 212)_ = 2.86, *p* = 0.0004), *Bacteroides* (F_(15, 212)_ = 3.16, *p* = 0.0001), *Turicibacter* (F_(15, 212)_ = 2.84, *p* = 0.0004), and *Pseudomonas* (F_(15, 212)_ = 1.79, *p* = 0.04) (Table S3). Farm significantly influenced the relative abundances of several of these taxa, including *Corynebacterium* (F_(5, 212)_ = 16.57, *p* < 0.0001), *Escherichia*–*Shigella* (F_(5, 212)_ = 2.96, *p* = 0.01), *Enterococcus* (F_(5, 212)_ = 10.67, *p* < 0.0001), *Turicibacter* (F_(5, 212)_ = 8.44, *p* < 0.0001), *Pseudomonas* (F_(5, 212)_ = 7.85, *p* < 0.0001), and *Staphylococcus* (F_(5, 212)_ = 3.10, *p* = 0.01). Within manure samples, the relative abundances of potential pathogens were significantly affected by the farm-by-sampling month interaction, including *Clostridium* (F_(15, 48)_ = 3.49, *p* = 0.0005), *Corynebacterium* (F_(15, 48)_ = 1.99, *p* = 0.04), *Dietzia* (F_(15, 48)_ = 2.99, *p* = 0.002), and *Turicibacter* (F_(15, 48)_ = 3.67, *p* = 0.0003) (Table S4). Overall, farm affected the relative abundances of most of the potential pathogenic taxa such as *Escherichia*–*Shigella* (F_(5, 48)_ = 3.38, *p* = 0.01), *Enterococcus* (F_(5, 48)_ = 5.67, *p* = 0.0003), *Acinetobacter* (F_(5, 48)_ = 4.83, *p* = 0.001), and *Bacteroides* (F_(5, 48)_ = 3.87, *p* = 0.005).

### 3.3. Bacterial Diversity and Community Composition

The bacterial Shannon diversity index varied among individual flies (range: 0.785–6.17) and among individual manure samples (range: 4.18–6.07). Overall, the Shannon diversity index was significantly lower in fly samples than in manure samples (t = −9.58, *p* < 0.001), and the sample type-by-farm interaction significantly affected the Shannon diversity index (F_(5, 296)_ = 8.20, *p* < 0.0001). Within farms, the mean Shannon diversity index in house fly samples was significantly lower than in manure samples [FLH (t = −7.08, *p* < 0.0001), FLU (t = −7.78, *p* < 0.0001), NCR (t = −2.71, *p* = 0.007) and TNP (t = −3.30, *p* = 0.001); (Figure 3b)]. Further, bacterial community composition in individual fly and manure samples within a farm varied by sample type (fly and manure) and sampling month. Although bacterial community composition in an individual house fly was distinctive, the PCoA analyses revealed that the individual flies sampled in the same month within a farm were clustered closer to each other than those that were collected from other months and manure samples (Figure S3).

The permutation-based analysis of variance (*adonis*) revealed that sample type and sampling month affected the bacterial community composition in fly and manure samples within a farm. For instance, sample type influenced the bacterial assemblage within FLH (pseudo-F_(1, 47)_ = 13.89, R^2^ = 0.20, *p* < 0.001), FLU (pseudo-F_(1, 45)_ = 15.84, R^2^ = 0.22, *p* < 0.001), NCC (pseudo-F_(1, 47)_ = 15.12, R^2^ = 0.20, *p* < 0.001), NCR (pseudo-F_(1, 47)_ = 29.50, R^2^ = 0.30, *p* < 0.001), TNL (pseudo-F_(1, 45)_ = 14.71, R^2^ = 0.21, *p* < 0.001), and TNP (pseudo-F_(1, 47)_ = 21.14, R^2^ = 0.28, *p* < 0.001). Also, sampling month influenced fly bacterial communities within each farm: FLH (pseudo-F_(3, 47)_ = 2.94, R^2^ = 0.13, *p* < 0.001), FLU (pseudo-F_(3, 45)_ = 4.15, R^2^ = 0.17, *p* < 0.001), NCC (pseudo-F_(3, 47)_ = 4.81, R^2^ = 0.19, *p* < 0.001), NCR (pseudo-F_(3, 47)_ = 7.48, R^2^ = 0.23, *p* < 0.001), TNL (pseudo-F_(3, 45)_ = 3.09, R^2^ = 0.13, *p* < 0.001), and TNP (pseudo-F_(3, 47)_ = 2.19, R^2^ = 0.0.09, *p* = 0.006).

### 3.4. Bacterial Generalist and Specialist Populations

Only 16.28% of house fly and 23.23% of manure sample OTUs were assigned to generalists, while 53.92% of house fly and 46.77% manure sample OTUs were specialists (Figure 3c). Interestingly, house fly and manure samples comprised 29.8% and 28.81% of OTUs designated as neutral (or non-significant), respectively. The most abundant generalists in fly samples included unclassified Bacteroidetes, Firmicutes, Ruminococcaceae, Lachnospiraceae, and Clostridia. Taxonomically similar bacterial taxa were assigned to generalists from manure samples as well. Specialists in fly samples were dominated by *Acinetobacter*, *Corynebacterium*, *Comamonas*, *Companilactobacillus*, *Escherichia*–*Shigella*, *Enterococcus*, unclassified Enterobacteriaceae, *Dysgonomonas*, *Vagococcus*, *Weissella*, *Providencia*, *Prevotella*, and *Turicibacter,* while manure samples were dominated by *Ruminobacter*, *Romboutsia*, *Clostridium* sensu stricto, *Paeniclostridium*, *Prevotella*, *Treponema*, *Turicibacter*, unclassified Lachnospiraceae, and Bacteroidales. At the higher taxonomic level (phylum), Firmicutes, Bacteroidetes, Proteobacteria, and Actinobacteria were the dominant specialist phyla in flies, while dominant specialists in manure samples were of the phyla Bacteroides and Firmicutes. Although variable in relative abundance, the dominant phyla of generalists in flies and/or manure samples were taxonomically similar to those of specialists.

Total relative abundances of generalists and specialists in house fly samples were significantly affected by farm-by-sampling month interaction (F _(15, 212)_ = 5.49 and 4.80, *p* < 0.0001 and <0.0001, respectively). Mean relative abundance of generalists in fly samples was significantly lower in July compared to May (t = −3.28, *p* = 0.007) and August (t = −3.04, *p* = 0.01) in NCC, while in the NCR farm, relative abundance was significantly higher in May than in June (t = 8.23, *p* < 0.0001), July (t = 8.27, *p* < 0.0001), and August (t = 8.14, *p* < 0.0001). Similarly, the relative abundance of specialists in fly samples was significantly higher in July than in May (t = 3.00, *p* = 0.01) and August (t = 3.06, *p* = 0.01) in NCC and in NCR, the relative abundance was significantly lower in May than in June (t = −7.49, *p* < 0.0001), July (t = −7.10, *p* < 0.0001), and August (t = −7.50, *p* < 0.0001). Farm significantly influenced the relative abundance of both generalists (F _(5, 212)_ = 20.92, *p* < 0.0001) and specialists (F _(5, 212)_ = 22.84, *p* < 0.0001) in fly samples. However, there were no differences in relative abundances of either generalists or specialists between farms within a farm location (Figure S4a,b). Relative abundances of generalists and specialists in manure samples were significantly influenced by farm-by-sampling month interaction (F _(15, 48)_ = 2.75 and 2.54, *p* = 0.004 and 0.007, respectively). The mean relative abundance of specialists in manure was significantly lower in May than in July (t = −2.87, *p* = 0.03) and August (t = −4.21, *p* = 0.0006) in FLH, while in the NCC farm, the relative abundance was significantly higher in July than in August (t = 3.12, *p* = 0.02). Similarly, the relative abundance of specialists in manure was significantly higher in May than in July (t = 3.02, *p* = 0.02) and August (t = 4.30, *p* = 0.0005) in FLH. Relative abundances of generalists and specialists in manure samples were significantly influenced by farm (F _(5, 48)_ = 26.69 and 27.14, *p* < 0.0001 and <0.0001, respectively). The relative abundance of generalists in manure samples was greater in the TNP than in the TNL farm (t = 5.05, *p* = 0.0001; Figure S4c), while the relative abundance of specialists in manures samples was lower in the TNP compared to the TNL farm (t = −6.35, *p* < 0.0001; Figure S4d).

## 4. Discussion

This study evaluated whether house fly bacterial communities reflected a major source of bacteria (i.e., manure) in dairy farms and whether these communities varied over time. We found, for the first time, that irrespective of farms, house flies from dairy farms captured all bacterial taxa that were present in bacterial communities of manure samples. However, within farms, some manure samples had unique taxa not found in co-located flies, although the relative abundances of these taxa were very low within the manure samples. Nonetheless, the majority of the fly’s microbiome comprised taxa from manure. Of note, although not all bacterial taxa (OTUs) were shared between house fly and manure samples within a farm, the shared bacterial taxa were also the most abundant in manure samples (>99%) within a farm, suggesting that bacterial communities carried by house flies at dairy cattle farms reflect their habitat. This phenomenon is not surprising since dairy farms sampled in this study housed cows living under confined conditions, providing continuous access to animals and their manure or other waste by co-located house flies.

In addition to the manure bacterial communities, house flies also carried unique taxa acquired from other sources, presumably animals, their feed, water, or the environment. Several bacterial taxa were differentially associated either with flies or with manure samples. For instance, *Corynebacterium*, *Staphylococcus*, and Enterobacteriaceae (unclassified), were differentially associated with fly samples. Several species of *Corynebacterium*, *Staphylococcus,* and Enterobacteriaceae can cause bovine mastitis [35,36], and house flies are potential transmitters of the mastitis pathogen *C. pseudotuberculosis* in milking cows [6]. Therefore, house flies may have acquired these taxa from mastitis-affected teats or milk. Of note, some surveyed dairy farms (FLH, NCC, and NCR) reported individual cattle with active mastitis being treated with antibiotics, suggesting that some of these pathogens may have been present at those locations. Other differentially abundant taxa were likely acquired from different microbial sources in the farm such as animal feed, water, or farm waste. Manure samples also harbored differentially abundant taxa like Clostridia, Bacteroidetes, and several others that have been previously reported from cattle rumen and/or dung [11,37]. For instance, bacterial communities characterized using shotgun and amplicon sequencing showed that taxa such as *Prevotella* (Prevotellaceae), *Bacteroides* (Bacteroidetes), *Ruminococcus* (Ruminococcaceae), Lachnospiraceae, and *Clostridium* (Clostridia) were the major bacterial taxa associated with cattle rumen, gut, and dung/manure [11,37].

Although both house fly and manure bacterial communities in individual farms shared a large number of OTUs, several bacterial taxa were differentially associated with each farm. Each farm management differed, and the farms were located large distances apart (being in TN, NC, and FL), enough to have climatic variation. Several prior studies conducted at animal farms and hospitals demonstrated that bacterial communities in flies varied between geographic locations [23,24]. Thus, some of the differentially associated bacteria observed across farms in our study were presumably due to the factors associated with local environmental biotic and abiotic factors, including climate and farm management. However, specific factors could not be identified in this study.

Adult house fly bacterial communities contained several potential human and animal pathogens, which concurs with our previous studies [10,24,25,27]. Most taxa were dominant in fly samples and were part of core bacterial communities and many taxa were highly prevalent in flies—together indicating that house flies may serve as sentinels for these taxa in dairy farms. Moreover, the abundances of such taxa were significantly higher in fly than manure samples, suggesting that flies may accumulate these potential pathogens and therefore pose a great risk to public and animal health. Bacterial pathogens associated with bovine respiratory disease (BRD; *Mannheimia*, *Pasteurella*, *Histophilus,* and *Mycoplasma*) and infectious bovine keratoconjunctivitis (IBK; *Moraxella*, *Mycoplasma*) were either not present or negligible in manure samples and when they were present in fly samples, their prevalence was very low. This finding is in accordance with our previous study in which we showed that house fly samples collected from beef farms carried BRD and IBK pathogens, but flies from dairy farms either did not carry these taxa or their prevalence was extremely low [24].

House flies and manure from cattle farms have highly diverse bacterial communities [11,12,15,16,17,20,21,22,24,27]. In our study, we observed greater overall (or average) bacterial diversity in manure than in house flies, and various factors could have contributed to these differences. For instance, among individual flies, there was greater variability in bacterial diversity (Shannon diversity index ranging from 0.785 to 6.17). This greater variability resulted in a lower average or overall bacterial diversity. Such variability in bacterial diversity among individual flies can be caused by a wide range of factors. Microbial source, age of the individual fly, body size, feeding or reproductive status, and physiology could have affected bacterial diversity [38]. In contrast, the manure samples had lower sample-to-sample variation in bacterial diversity, potentially because manure was collected from homogenized sources (piles, scrapes) that represented herds of animals. Further, no significant changes occurred in dairy cattle or farm management methods between sampling dates, which presumably contributed to minimal environmental variation. Thus, we observed a lower effect of sampling month on bacterial diversity in manure than in flies. Furthermore, bacterial community composition in manure samples was more closely clustered together than that of individual fly samples within a farm. Significant effects of sampling month on bacterial community composition of house fly and manure samples within farms suggests that slightly different weather conditions and/or other undetermined biotic or abiotic factors may have influenced the abundance of several bacterial taxa in the farms, which subsequently altered the community composition within a farm and was subsequently reflected in fly samples.

In theory, bacterial species with the ability to use a broad range of resources (e.g., generalists) could maintain large populations with low phylogenetic variability, where few species thrive and occupy a broad ecological niche/habitat. In contrast, specialists restricted to utilizing specific resources would have lower population densities with larger phylogenetic variations because they are usually restricted to a specific niche or habitat. In specialist insect hosts, such as termites and bees, specialized gut bacterial communities establish symbiotic relationships where bacterial specialists convert complex organic matter into low molecular weight compounds such as proteins and sugars that can be utilized as energy sources by the host [39,40]. Consequently, specialist hosts harbor few bacterial specialists but with a high population density. In contrast, house flies feed on a wide range of decomposing organic matter and thrive in diverse habitats. Additionally, house flies carry bacteria in their gut transiently as the gut is lined with a peritrophic matrix which restricts the bacteria to the lumen, and inhibits colonization of the gut epithelium and other tissues [1]. Therefore, we expected to detect a high diversity and high relative abundance of bacterial generalist species, and a low diversity and low relative abundance of specialist species in house flies. In contrast, we actually determined that house flies carried highly diverse and highly abundant specialist species, and had a low abundance yet diverse generalist species. This phenomenon can be attributed to the source of the microbes they acquire, predominantly cattle manure, which itself is mostly cattle rumen/gut bacteria. Dairy cattle rumen or lower gut bacterial communities are composed of more bacterial specialists than generalists [41]. These microbes are shed in manure and acquired by house flies, who regularly practice coprophagy [42,43]. Hence, the high abundance and diversity of specialists in house flies reflects their main source of bacteria, which is dairy cattle manure.

## 5. Conclusions

In summary, adult house flies from dairy cattle farms carried highly diverse bacterial communities and likely acquired these communities from manure and/or animals within the farm. House fly bacterial communities primarily comprised specialist species likely sourced from cattle manure, and these taxa were of veterinary and anthropogenic importance. Thus, house flies pose a risk of dissemination of these taxa among cattle and the surrounding environments, which may include residential areas. The findings of this study highlight not only the importance of house flies as carriers and sources of microbial threats, but also their utility as sentinels for microbes acquired from animals, manure, and the environment.

## Data Availability

The data that support the findings of this study are included in the article and Supplementary Materials. Also, raw sequence data are available at the National Center for Biotechnology Information Sequence Read Archive under the BioProject number PRJNA1070634.

## Supplementary Materials

The following supporting information can be downloaded at: https://www.mdpi.com/article/10.3390/insects15090730/s1, Figure S1: Bacterial communities (observed operational taxonomic units, OTUs) in house fly and manure samples within a farm. Overlapping sections represent the shared bacterial communities, observed number of OTUs, between house fly and manure samples, and non-overlapping sections represent OTUs unique to the sample types (house fly or manure) on dairy farms: (a) FLH, (b) FLU, (c) TNL (upper panel) and (d) TNP, (e) NCC, (f) NCR (lower panel).; Figure S2: Prevalence and abundance profiles of potential bacterial pathogens in house fly and manure samples associated with dairy farms. (a) Prevalence (%; n = 40 per farm, except FLU and TNL, n = 38) and abundance (row z-score of mean relative abundance; n = 40, except FLU and TNL, n = 38) in house fly samples. (b) Prevalence (%; n = 12 per farm) and abundance (row z-score of mean relative abundance; n = 12 per farm) in manure samples.; Figure S3: Bacterial community composition in house flies associated with dairy farms. The first and second principal coordinate analysis (PCoA) axes show the bacterial community composition in individual house fly and manure samples collected in May, June, July, and August in (a) FLH, (b) FLU, (c) NCC (upper panel) and (d) NCR, (e) TNL, (f) TNP (lower panel) dairy farms.; Figure S4: Generalist and specialist bacterial communities in house fly and manure samples from dairy farms. Relative abundance of bacterial generalists in (a) fly and (c) manure samples and specialists in (b) fly and (d) manure samples for each farm (see main text for descriptions). Plots depict the median and interquartile ranges (25th–75th percentile; boxes) with whiskers representing upper and lower values, respectively, with outlier points. Asterisks (***) indicate significant differences between farms within a farm’s geographic location (state) at *p* < 0.0001 and ns indicates non-significance. All farms had n = 40 samples (except FLU and TNL n = 38) of house flies and n = 12 manure samples.; Table S1: Metadata associated with sampling sites (farms); Table S2: Effects of farm and sample type on relative abundances of potential pathogens in house fly and manure samples associated with dairy farms; Table S3: Effects of farm and sampling month on relative abundances of potential pathogens in house fly samples associated with dairy cattle farms; Table S4: Effects of farm and sampling month on relative abundances of potential pathogens in manure samples associated with dairy cattle farms.
