# Supplementary material for: Bacterial Communities of House Flies from Dairy Farms Highlight Their Role as Reservoirs, Disseminators, and Sentinels of Microbial Threats to Human and Animal Health
Source: Insects. 2024 Sep 22;15(9):730. doi: 10.3390/insects15090730 (PMC11432648; doi:10.3390/insects15090730)
Supplement: Supplementary file 1 [file insects-15-00730-s001.zip › insects-3181988-supplementary.pdf]

Supplementary Materials

Bacterial Communities of House Flies from Dairy Farms Highlight Their Role as Reservoirs, Disseminators, and Sentinels of Microbial Threats to Human and Animal Health

Saraswoti Neupane[1], Yoonseong Park[1], D. Wes Watson[2], Rebecca T. Trout Fryxell[3], Edwin R. Burgess IV[4], Dana Nayduch[5]

[1]Department of Entomology, Kansas State University, Manhattan, KS 66506, USA

[2]Department of Entomology and Plant Pathology, North Carolina State University, Raleigh, NC 27695, USA

[3]Department of Entomology and Plant Pathology, University of Tennessee, Knoxville, TN 37996, USA

[4]Department of Entomology and Nematology, University of Florida, Gainesville, FL 32611, USA

[5]USDA-ARS, Center for Grain and Animal Health Research, Arthropod-Borne Animal Diseases Research Unit, Manhattan, KS 66502, USA

Supplementary Figures

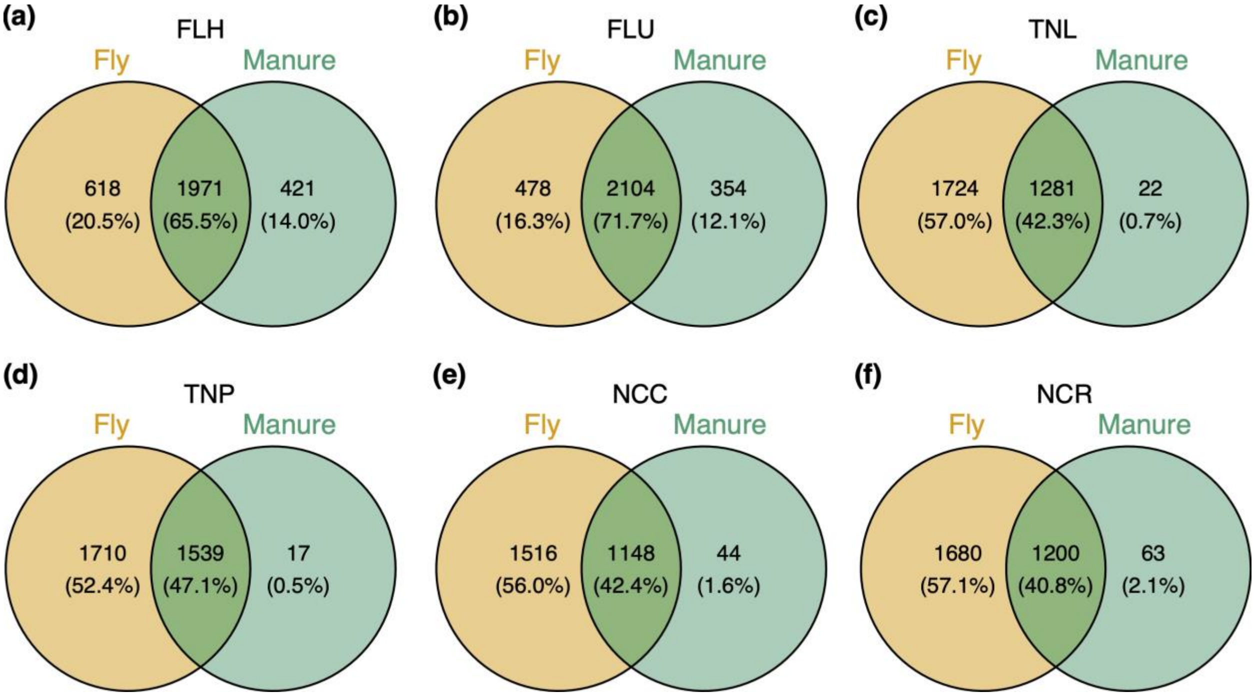

Figure S1: Bacterial communities (observed operational taxonomic units, OTUs) in house fly and manure samples within a farm. Overlapping sections represent the shared bacterial communities, observed number of OTUs, between house fly and manure samples, and non-overlapping sections represent OTUs unique to the sample types (house fly or manure) at dairy farms: (a) FLH, (b) FLU, (c) TNL (upper panel) and (d) TNP, (e) NCC, (f) NCR (lower panel).

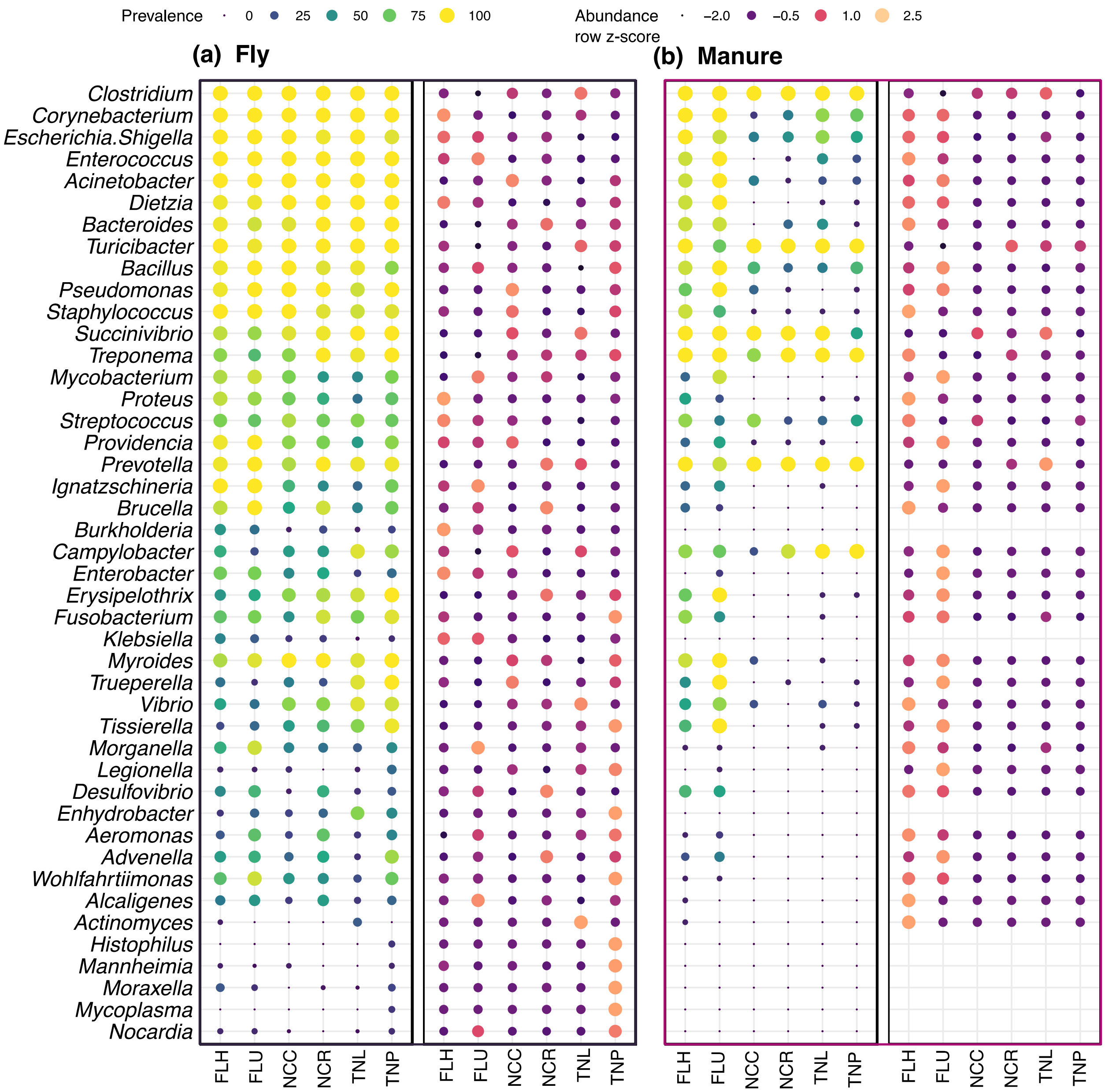

Figure S2: Prevalence and abundance profiles of potential bacterial pathogens in house fly and manure samples associated with dairy farms. (a) prevalence (%; n = 40 per farm except FLU and TNL, n = 38) and abundance (row z-score of mean relative abundance; n = 40, except FLU and TNL, n = 38) in house fly samples. (b) prevalence (%; n = 12 per farm) and abundance (row z-score of mean relative abundance; n = 12 per farm) in manure samples.

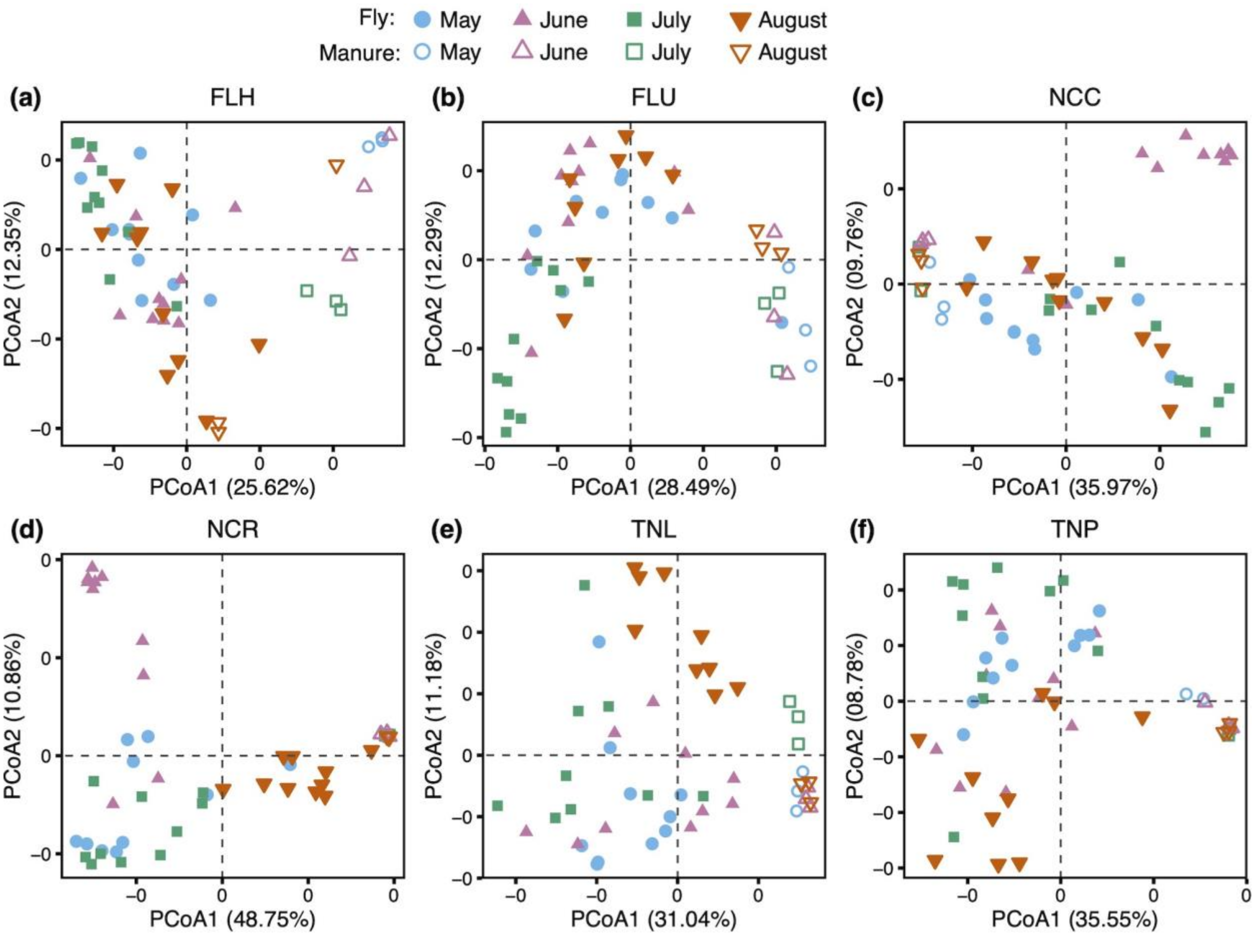

Figure S3: Bacterial community composition in house flies associated with dairy farms. The first and second Principal coordinate analysis (PCoA) axes show the bacterial community composition in individual house fly and manure samples collected in May, June, July, and August at (a) FLH, (b) FLU, (c) NCC (upper panel) and (d) NCR, (e) TNL, (f) TNP (lower panel) dairy farms.

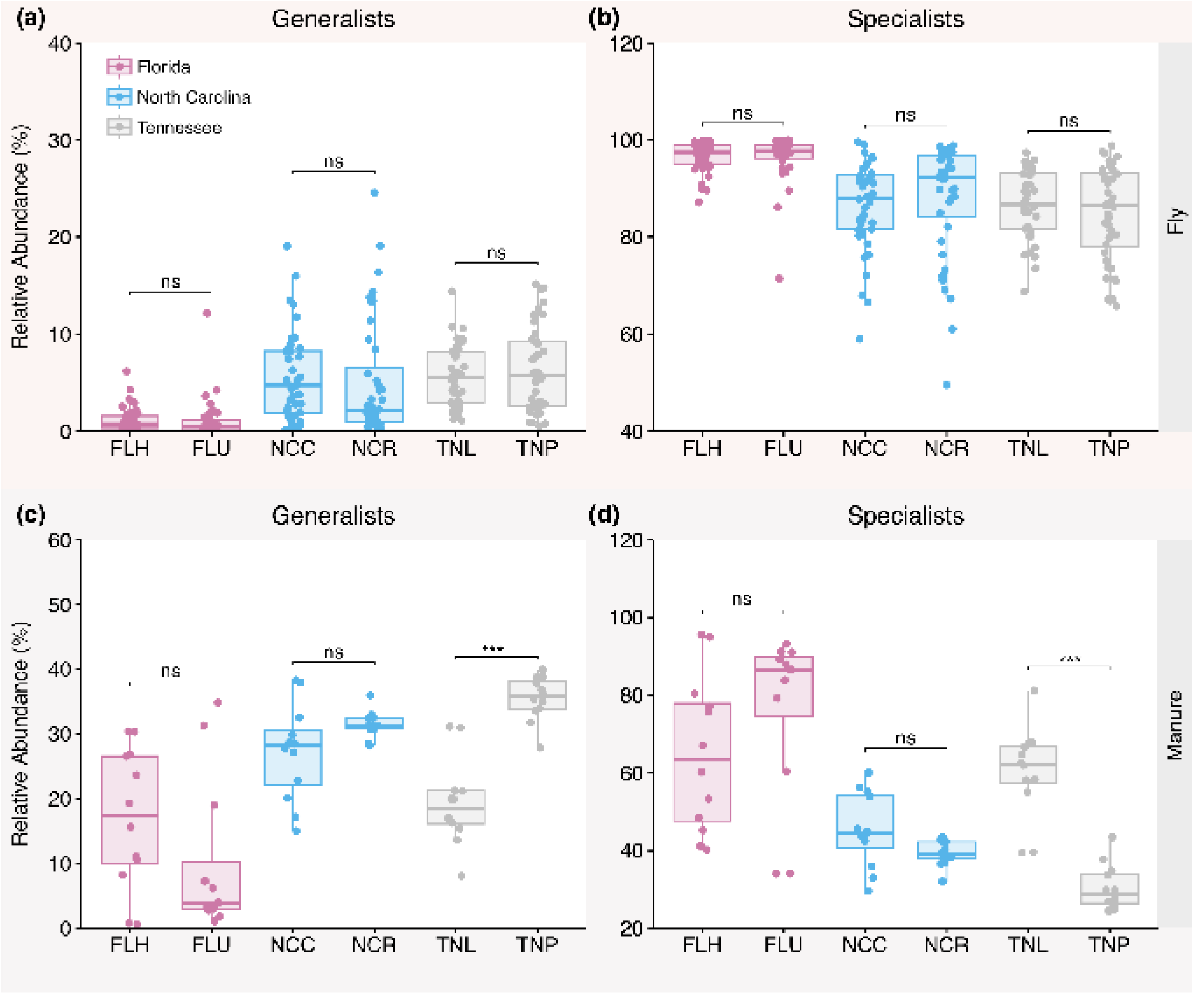

Figure S4: Generalist and specialist bacterial communities in house fly and manure samples from dairy farms. Relative abundance of bacterial generalists in (a) fly and (c) manure samples and specialists in (b) fly and (d) manure samples for each farm (see main text for descriptions). Plots depict the median and interquartile ranges (25th – 75th percentile; boxes) with whiskers representing upper and lower values, respectively with outlier points. Asterisks (***) indicate significant differences between farm within a farm's geographic location (state) at p <0.0001 and ns indicates non-significant. All farms had n = 40 samples (except FLU and TNL n = 38) of house flies and n = 12 manure samples.

Supplementary Tables

Table S1: Metadata associated with sampling sites (farms).

State	Farm	Latitude, Longitude	Cattle breed; Age & Sex	Diet at pen	Lot capacity (head)	Manure management	Fly management	Antibiotic use	Other animals nearby	Sampling Date (year 2021)
Florida	FLH	29.61 N, 82.09 W	Holstein, Jersey and cross; mixed male and female	Total mix ration (TMR)	825	Spread, dry scrape	Yes	Yes	Yes (donkeys)	May 7, June 9, July 21, August 24
Florida	FLU	29.79 N, 82.41 W	Holstein, Jersey and cross; adult females	Grain / TMR (silage)	700/500	Piled in pen	Yes	Yes	Yes (beef cattle)	May 7, June 9, July 21, August 24
North Carolina	NCC	35.92 N 79.12 W	Jersey; mixed	Pasture, hay, roughage	60	Piled nearby, spread	Yes	Yes	Yes (pigs)	May 18, June 11, July 7, August 10
North Carolina	NCR	35.73 N 78.68 W	Holstein, Jersey, Brown Swiss; mixed	Grain, silage, roughage and pasture	380	pile nearby, pile in pen, spread, compost	Yes	Yes	Yes (beef, poultry, swine within a mile)	May 17, June 8, July 6, August 9
Tennessee	TNL	35.66 N, 84.20 W	Holstein; heifers and cows	Grain silage	1500	Spread, hauled away	Yes	Yes	No	May 20, June 24, July 20, August 26
Tennessee	TNP	35.68 N, 84.39 W	Holstein; mixed female	Grain silage	2000+	Spread	Yes	No	No	May 20, June 24, July 20, August 24

Table S2. Effects of farm and sample type on relative abundances of potential pathogens in house fly and manure samples associated with dairy farms.

Taxon	Farm		Sample		Farm:Sample	
	$F_{(5, 296)}$	p-value	$F_{(1, 296)}$	p-value	$F_{(5, 296)}$	p-value
Clostridium	8.79	**<0.0001**	86.78	**<0.0001**	7.88	**<0.0001**
Corynebacterium	18.01	**<0.0001**	39.52	**<0.0001**	3.02	**0.0113**
Escherichia-Shigella	2.95	**0.0129**	14.81	**0.0001**	0.87	0.5029
Enterococcus	10.56	**<0.0001**	25.71	**<0.0001**	2.19	0.0551
Acinetobacter	1.67	0.1407	15.08	**0.0001**	1.50	0.1895
Dietzia	2.43	**0.0350**	5.38	**0.0210**	0.39	0.8543
Bacteroides	1.35	0.2436	8.46	**0.0039**	2.02	0.0760
Turicibacter	9.10	**<0.0001**	24.36	**<0.0001**	2.73	**0.0196**
Bacillus	0.72	0.6118	4.50	**0.0347**	0.17	0.9732
Pseudomonas	6.15	**<0.0001**	10.27	**0.0015**	2.97	**0.0125**
Staphylococcus	3.19	**0.0080**	8.27	**0.0043**	0.97	0.4380
Succinivibrio	33.38	**<0.0001**	100.14	**<0.0001**	25.31	**<0.0001**
Treponema	3.57	**0.0038**	134.73	**<0.0001**	12.57	**<0.0001**
Mycobacterium	0.86	0.5074	2.56	0.1110	0.19	0.9647
Proteus	2.49	**0.0313**	1.54	0.2151	0.74	0.5908
Streptococcus	0.77	0.5726	2.62	0.1069	0.20	0.9608
Providencia	2.47	**0.0329**	4.52	**0.0343**	0.75	0.5892
Prevotella	23.25	**<0.0001**	22.70	**<0.0001**	10.63	**<0.0001**
Ignatzschineria	6.46	**<0.0001**	7.30	**0.0073**	2.00	0.0790
Brucella	6.03	**<0.0001**	12.83	**0.0004**	1.83	0.1063
Burkholderia	2.28	0.0468	2.03	0.1552	0.69	0.6333
Campylobacter	0.70	0.6253	7.09	**0.0082**	4.39	**0.0007**
Enterobacter	1.17	0.3222	2.55	0.1114	0.35	0.8799
Erysipelothrix	3.93	**0.0018**	3.66	0.0568	9.38	**<0.0001**
Fusobacterium	3.51	**0.0043**	4.13	**0.0430**	1.14	0.3405
Klebsiella	1.05	0.3906	5.48	**0.0198**	0.32	0.9005
Myroides	2.52	**0.0298**	8.52	**0.0038**	2.25	**0.0498**
Trueperella	2.17	0.0578	2.59	0.1089	1.56	0.1707
Vibrio	1.38	0.2312	1.87	0.1722	0.87	0.5037
Tissierella	8.18	**<0.0001**	18.10	**<0.0001**	37.33	**<0.0001**
Morganella	2.40	0.0376	4.47	0.0354	0.75	0.5897
Legionella	1.76	0.1204	3.50	0.0624	0.67	0.6441
Desulfovibrio	4.88	**0.0003**	1.21	0.2731	2.06	0.0708
Enhydrobacter	1.95	0.0857	1.25	0.2651	0.59	0.7105
Aeromonas	0.59	0.7099	2.94	0.0874	0.18	0.9682

Advenella	1.92	0.0903	9.44	**0.0023**	0.61	0.6937
Wohlfahrtiimonas	3.93	**0.0018**	3.86	0.0503	1.18	0.3165
Alcaligenes	1.39	0.2268	3.53	0.0613	0.45	0.8129
Actinomyces	2.14	0.0602	0.69	0.4080	0.69	0.6351
Histophilus	2.26	**0.0483**	0.68	0.4087	0.68	0.6380
Mannheimia	0.98	0.4329	0.66	0.4159	0.29	0.9157
Moraxella	1.04	0.3949	0.41	0.5221	0.31	0.9051
Mycoplasma	3.48	**0.0045**	1.05	0.3057	1.05	0.3897
Nocardia	0.89	0.4898	0.72	0.3966	0.27	0.9294

Table S3: Effects of farm and sampling month on relative abundances of potential pathogens in house fly associated with dairy cattle farms.

Pathogen	Farm		Month		Farm:Month	
	$F_{(5, 212)}$	p-value	$F_{(3, 212)}$	p-value	$F_{(15, 212)}$	p-value
Clostridium	2.12	0.0643	5.97	**0.0006**	1.46	0.1230
Corynebacterium	16.57	**<0.0001**	1.68	0.1731	1.71	0.0509
Escherichia-Shigella	2.96	**0.0133**	0.67	0.5728	0.97	0.4861
Enterococcus	10.67	**<0.0001**	1.66	0.1763	2.30	**0.0047**
Acinetobacter	2.17	0.0590	3.45	**0.0174**	2.15	**0.0091**
Dietzia	1.78	0.1191	7.17	**0.0001**	2.86	**0.0004**
Bacteroides	2.44	**0.0356**	1.53	0.2086	3.16	**0.0001**
Turicibacter	8.44	**<0.0001**	13.21	**<0.0001**	2.84	**0.0004**
Bacillus	0.71	0.6151	2.58	0.0543	1.03	0.4226
Pseudomonas	7.85	**<0.0001**	9.88	**<0.0001**	1.79	0.0375
Staphylococcus	3.10	**0.0101**	0.71	0.5453	0.61	0.8688
Succinivibrio	16.17	**<0.0001**	6.52	**0.0003**	5.49	**<0.0001**
Treponema	9.23	**<0.0001**	3.11	**0.0272**	3.18	**0.0001**
Mycobacterium	0.82	0.5338	0.24	0.8678	1.35	0.1738
Proteus	2.61	0.0257	1.75	0.1571	1.59	0.0785
Streptococcus	0.75	0.5855	0.66	0.5757	1.10	0.3555
Providencia	2.42	**0.0369**	0.70	0.5535	0.56	0.9023
Prevotella	11.59	**<0.0001**	4.38	**0.0051**	1.79	**0.0380**
Ignatzschineria	7.02	**<0.0001**	2.11	0.0997	1.64	0.0664
Brucella	9.89	**<0.0001**	9.69	**<0.0001**	8.62	**<0.0001**
Burkholderia	2.65	**0.0240**	3.88	**0.0100**	2.63	**0.0012**
Campylobacter	1.18	0.3222	1.78	0.1524	1.26	0.2328
Enterobacter	1.22	0.2988	1.29	0.2792	1.43	0.1366
Erysipelothrix	9.10	**<0.0001**	8.76	**<0.0001**	4.08	**<0.0001**
Fusobacterium	4.35	**0.0009**	1.91	0.1286	3.83	**<0.0001**

Klebsiella	1.05	0.3905	1.71	0.1651	0.81	0.6652
Myroides	3.90	**0.0021**	3.14	**0.0264**	3.78	**<0.0001**
Trueperella	3.34	**0.0064**	2.95	**0.0338**	4.59	**<0.0001**
Vibrio	1.74	0.1278	0.93	0.4249	1.57	0.0853
Tissierella	18.70	**<0.0001**	3.44	**0.0177**	2.84	**0.0005**
Morganella	2.39	**0.0394**	0.33	0.8048	1.05	0.4056
Legionella	1.99	0.0820	1.37	0.2531	1.93	**0.0216**
Desulfovibrio	5.01	**0.0002**	0.57	0.6338	1.22	0.2568
Enhydrobacter	1.94	0.0885	0.69	0.5570	0.80	0.6774
Aeromonas	0.61	0.6899	0.60	0.6159	1.48	0.1138
Advenella	2.30	**0.0464**	0.59	0.6196	3.66	**<0.0001**
Wohlfahrtiimonas	4.65	**0.0005**	3.76	**0.0117**	3.17	**0.0001**
Alcaligenes	1.48	0.1980	0.85	0.4666	1.41	0.1447
Actinomyces	2.32	0.0446	2.03	0.1113	1.86	**0.0290**
Histophilus	2.31	**0.0455**	1.05	0.3705	1.04	0.4138
Mannheimia	0.99	0.4231	0.74	0.5297	1.09	0.3698
Moraxella	1.05	0.3876	0.99	0.3974	0.94	0.5193
Mycoplasma	3.58	**0.0040**	1.20	0.3097	1.17	0.2935
Nocardia	0.88	0.4934	0.72	0.5419	0.99	0.4718

Table S4: Effects of farm and sampling month on relative abundances of potential pathogens in manure samples associated with dairy cattle farms.

**Taxon**	**Farm**		**Month**		**Farm:Month**	
	$\mathbf{F_{(5, 48)}}$	**p-value**	$\mathbf{F_{(3, 48)}}$	**p-value**	$\mathbf{F_{(15, 48)}}$	**p-value**
Clostridium	19.16	**<0.0001**	2.44	0.0762	3.49	**0.0005**
Corynebacterium	12.09	**<0.0001**	4.33	**0.0089**	1.99	0.0368
Escherichia-Shigella	3.34	**0.0114**	1.32	0.2788	0.94	0.5301
Enterococcus	5.67	**0.0003**	0.88	0.4572	0.85	0.6161
Acinetobacter	4.83	**0.0012**	0.72	0.5445	0.76	0.7143
Dietzia	6.19	**0.0002**	5.27	**0.0032**	2.99	**0.0020**
Bacteroides	3.87	**0.0050**	1.47	0.2353	1.81	0.0616
Turicibacter	8.21	**<0.0001**	4.23	**0.0099**	3.67	**0.0003**
Bacillus	6.49	**0.0001**	0.18	0.9101	0.82	0.6523
Pseudomonas	6.85	**0.0001**	1.94	0.1356	1.46	0.1571
Staphylococcus	1.72	0.1476	0.92	0.4400	0.95	0.5152
Succinivibrio	25.53	**<0.0001**	1.00	0.4005	1.83	0.0575
Treponema	6.69	**0.0001**	5.25	**0.0033**	3.29	**0.0009**
Mycobacterium	36.16	**<0.0001**	4.23	**0.0098**	2.56	**0.0070**
Proteus	5.31	**0.0006**	1.44	0.2430	1.31	0.2351

Streptococcus	5.53	**0.0004**	0.36	0.7793	2.43	**0.0102**
Providencia	2.70	**0.0312**	1.91	0.1405	1.52	0.1344
Prevotella	23.38	**<0.0001**	3.47	**0.0232**	1.33	0.2216
Ignatzschineria	3.17	**0.0150**	1.54	0.2155	1.60	0.1108
